# *“It's Changed My Mind-Set About the Idea of Motherhood*”: An Online Perinatal CFT Group Service Evaluation

**DOI:** 10.1192/bjo.2024.467

**Published:** 2024-08-01

**Authors:** Joanna Douzenis, Mahnoor Lashari

**Affiliations:** West London NHS, London, United Kingdom

## Abstract

**Aims:**

We aim to systematically document our reflections regarding the establishment of a perinatal-focused Compassion Focused Therapy (CFT) group within an expanding service. It aims to highlight specific outcomes and client experiences resulting from group completion.

**Methods:**

Synthesizing information from established CFT Group protocols across various National Health Service (NHS) contexts, scholarly investigations, and our CFT training, this study instituted a 10-week perinatal-CFT group intervention. Recruitment targeted individuals already engaged in our services, resulting in the referral and screening of eleven potential participants. Nine eligible individuals provided informed consent, with seven successfully completing the program. Assessments, including the Clinical Outcomes in Routine Evaluation (CORE-10), Postpartum Bonding Questionnaire (PBQ), The Forms of Self-criticizing/Attacking & Self-reassuring Scale (FSCRS), and Maternal Antenatal Attachment Scale (MAAS), were administered pre- and post-group. Quantitative findings were analysed and compared, supplemented by qualitative insights distilled from thematic analyses of feedback forms and post-group reviews with each participant.

**Results:**

Though we had a small number of participants (n = 4) who completed the pre and post measures and the post group review, we received overall positive feedback for the group intervention. During the post group review and from their feedback forms, participants expressed the value of the group experience and found the discussions and exploration of CFT concepts to be helpful in reflecting on their self-critical thoughts.

On the Core-10, there was a reliable and clinically significant change for 75% of participants. Two participants completed the PBQ, and both showed a reliable but not clinically significant change in scores. We had one antenatal client who showed a reliable but not clinically significant change on the MAAS.

The FSCRS comprises three scales: Inadequate Self (IS), Reassured Self (RS), and Hated Self (HS). On the IS subscale, a reliable and clinically significant change was observed for 75% of participants. The HS subscale showed a reliable change but lacked clinical significance for 50% of participants. No reliable change was observed in the RS scale for any participant.

**Conclusion:**

While the study's results are not generalizable due to the small sample size, positive feedback suggests the well-received nature of online perinatal CFT groups. Despite a preliminary evidence base, this paper contributes reflections and experiences, highlighting the potential effectiveness of online CFT groups in the perinatal period. These findings underscore the need for further research and exploration in this promising therapeutic approach.

